# Cushing’s disease across the lifespan: a case-based clinical review

**DOI:** 10.20945/2359-4292-2026-0043

**Published:** 2026-04-20

**Authors:** Elisabeth Nowak, Júnia Ribeiro de Oliveira Longo Schweizer, Margaret Cristina da Silva Boguszewski, Martin Reincke, Cesar Luiz Boguszewski

**Affiliations:** 1 Department of Medicine IV, LMU University Hospital, LMU Munich, Munich, Germany; 2 Departamento de Pediatria, Serviço de Endocrinologia e Metabologia (SEMPR), Universidade Federal do Paraná, Curitiba, PR, Brasil; 3 Departamento de Medicina Interna, Serviço de Endocrinologia e Metabologia (SEMPR), Universidade Federal do Paraná, Curitiba, PR, Brasil

**Keywords:** Pituitary, cortisol, children, adults, elderly, individualized care

## Abstract

Cushing’sdisease (CD), caused by an adrenocorticotropin-secreting pituitary adenoma, is a rare but severe endocrine disorder associated with high cardiometabolic morbidity and mortality. Diagnosis is challenging as many symptoms are nonspecific, and biochemical or imaging results may be inconclusive, contributing to substantial diagnostic delay. Although the core features of CD are consistent across the lifespan, certain clinical manifestations of chronic hypercortisolism vary from childhood through older age, reflecting differences in growth, puberty, metabolism, and comorbidity burden. In children, impaired growth coupled with weight gain is most prominent, whereas adolescents often present with pubertal disturbances and psychological or academic difficulties. Adults typically exhibit the classic Cushingoid features such as round face, plethora, and central obesity, along with metabolic and reproductive complications. Older individuals typically present with frailty, sarcopenia, fractures, and cognitive decline. Age also influences the interpretation of endocrine tests, the accuracy of pituitary magnetic resonance imaging, the role of inferior petrosal sinus sampling, perioperative risks, and the long-term impact of remission or persistent disease. Given this context, this narrative review used five representative clinical vignettes (pediatric, adolescent, adult female, adult male, and elderly) to illustrate how the presentation, diagnostic evaluation, and management of CD vary across the lifespan. Each case was paired with a structured synthesis of current evidence, highlighting both shared principles and age-specific nuances essential for timely diagnosis, appropriate treatment selection, and effective long-term multidisciplinary care. Understanding age-related differences is crucial to improving outcomes and reducing the substantial morbidity and mortality associated with CD throughout the lifespan.

## INTRODUCTION

Cushing’s disease (CD) is caused by chronic cortisol excess resulting from an adrenocorticotropin (ACTH)-secreting pituitary adenoma ^(1,2)^. With a global incidence of approximately 0.24 (95% confidence interval [CI] 0.15-0.33) cases per 100,000 person-years ^(3)^ and a substantial diagnostic delay ^(4)^, CD remains a challenging condition to recognize. Uncontrolled hypercortisolism has profound multisystem consequences, leading to increased cardiometabolic morbidity and mortality if not rapidly treated ^(1)^.

**Figure 1:**
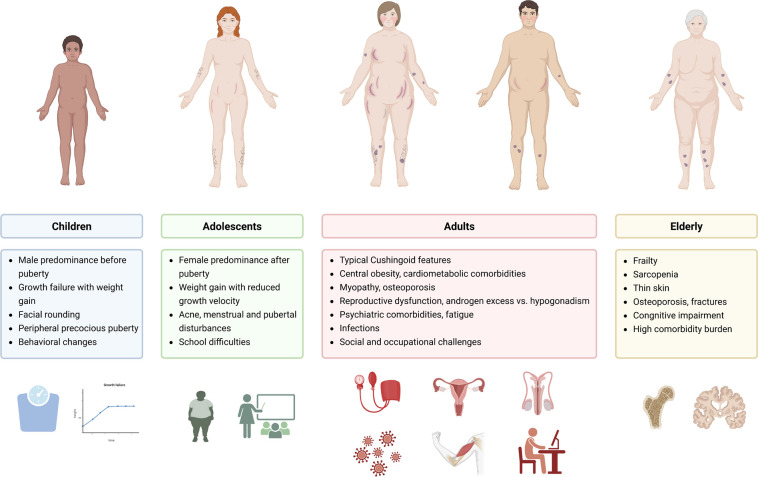
Key clinical features of Cushing’s syndrome across the lifespan. Created with biorender.

Although the core features of CD are similar at all ages, each age group exhibits characteristic clinical differences reflecting their developmental stage (**Figure 1**). In young children, growth failure and weight gain predominate, while adolescents often experience pubertal disturbances, weight gain, and menstrual changes ^(5,6)^. Among adults, metabolic and reproductive complications are common, whereas older individuals frequently develop frailty, sarcopenia, fractures, or cognitive decline, which may be misattributed to normal aging or comorbidities ^(1,7)^. Recognizing these differences is crucial for interpreting diagnostic tests and selecting appropriate treatment strategies. For example, dynamic test interpretation differs in children, fertility considerations are key for reproductive-age patients, and risk-benefit assessments require particular caution in older adults. Long-term complications and recurrence risk also differ across the lifespan, highlighting the need for age-tailored management ^(1,5)^.

This narrative review provides a structured, case-based overview of CD across the age spectrum - from childhood through adolescence, adulthood, and older age. For each age group, we present a representative clinical vignette followed by a concise synthesis of current evidence regarding etiology, clinical and biochemical presentation, diagnostic strategies (**Table 1**), and therapeutic considerations (**Table 2**). By highlighting both shared and age-specific patterns of CD, we aim to support clinicians in recognizing and managing this complex condition at every stage of life.

**Table 1:** Diagnostic considerations according to lifespan

Domain	Children	Adolescents	Adults	Elderly
Subtype frequency ^(14,63,69,70)^ Pituitary Adrenal Ectopic	~70-80% Predominant in very young children Very rare	~70-80% ~15-20% Very rare	~60-70% ~20-30% ~5-10%	~69% ~25% ~6%
Key diagnostic challenges	Physiologic variability of cortisol Difficulties in cooperation for testing	Overlap with normal puberty (acne, mood, weight changes), non-neoplastic hypercortisolism	Wide comorbidity spectrum (e.g., metabolic syndrome, depression) can mask early disease	Symptoms may be misattributed to aging Multimorbidity and polypharmacy complicate interpretation
Considerations for dynamic testing	Weight-specific cortisol cut-offs 24h-UFC correction for body surface area	Weight-specific cortisol cut-offs Need to distinguish CD from non-neoplastic hypercortisolism	Higher specificity of LNSC and LDDST	Reduced renal function can affect 24h-UFC Exogenous glucocorticoid therapy can complicate diagnosis
Pituitary imaging	Microadenomas often <3-4 mm MRI-negative in ~40-50%	Microadenomas often <3-4 mm MRI-negative in ~40-50%	Higher MRI detection rates Microadenomas more frequent than macroadenomas	Cerebral atrophy may reduce contrast resolution
Use of IPSS	Selective use in MRI-negative or discordant biochemistry	Selective use in MRI-negative or discordant biochemistry	Standard practice when imaging is negative or adenoma < 6-10 mm	Higher procedural risk depending on comorbidities but often still indicated

CD: Cushing’s disease; IPSS: inferior petrosal sinus sampling; LDDST: low dose (1 mg) dexamethasone suppression test; LNSC: late night salivary cortisol; MRI: magnet resonance imaging; 24h-UFC: 24h urinary free cortisol.

**Table 2:** Therapeutic considerations according to lifespan

Domain	Children	Adolescents	Adults	Elderly
Surgical considerations	Requires experienced pediatric pituitary surgeon	Requires experienced pediatric pituitary surgeon	Established first-line therapy	Higher perioperative risk, individualized decision-making
Medical therapy	Reserved for persistent/recurrent disease	Reserved for persistent/recurrent disease	Standard second-line options (steroidogenesis inhibitors, pituitary-directed drugs) Individual choice depending on severity, comorbidities, and gender	Polypharmacy and comorbidities guide choice and dosing
Long-term issues	Catch-up growth Pubertal progression Bone health	Psychosocial support School performance Fertility	Cardiometabolic Psychological Social and occupational impact Risk of recurrence	Frailty Cognitive decline Falls and fracture prevention

## INFANTS AND PREPUBERTAL CHILDREN (0-10 YEARS)

### Case vignette

**Figure 2:**
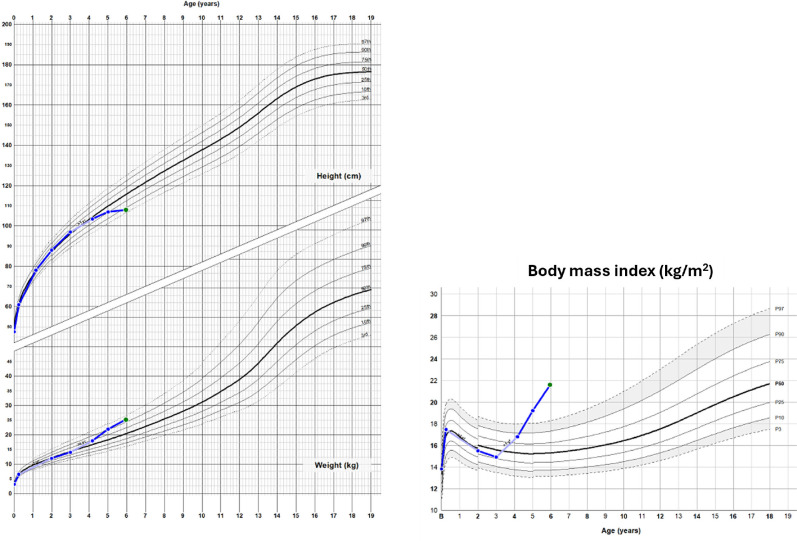
Growth charts of a 6-year-old boy with Cushing’s disease showing growth failure with simultaneous weight gain and increased BMI.

A 6-year-old boy with 18 months of growth deceleration, central weight gain, facial rounding, and new-onset hypertension. His parents reported mood swings and decreased academic performance, prompting an ongoing investigation for autism. On examination, his height was -1.6 standard deviation score (SDS), weight +1.5 SDS, and BMI +3.4 SDS (**Figure 2**). Biochemical testing showed repeatedly elevated 24-hour urinary free cortisol (24h-UFC) and inadequate suppression of morning serum cortisol on low-dose dexamethasone. ACTH levels were inappropriately normal to high. Pituitary magnetic resonance imaging (MRI) revealed a left-sided 4-mm microadenoma. Given the concordant MRI findings and the rarity of ectopic ACTH secretion in this age group, the multidisciplinary team proceeded directly to surgery without further confirmatory testing. Transsphenoidal surgery confirmed an ACTH-secreting microadenoma; postoperative glucocorticoid replacement was required. On follow-up, hypertension resolved, and both growth velocity and mood improved.

### Mini-review

Cushing’s disease is rare in childhood, with an incidence of approximately 0.5 new cases per million children per year, roughly 10% of the adult rate ^(5)^. Corticotroph microadenomas are the most common pituitary tumors in childhood, comprising ~55% of adenomas in ages 0-11 and ~30% in ages 12-17 ^(5)^. Cushing’s disease is exceedingly rare below the age of 5, in whom adrenal causes predominate ^(8)^. Marked male predominance (~70%) is observed before puberty, with boys demonstrating higher ACTH concentrations and more aggressive features ^(5)^. Growth failure with simultaneous weight gain is the hallmark symptom, with height SDS decreasing while body mass index (BMI) increases ^(6)^ (**Figure 2**). Unlike adults, children with CD rarely have myopathy, sleep, memory disturbances, or skin striae ^(9)^. Current pediatric guidelines recommend screening only when unexplained weight gain is accompanied by decreased height velocity or height percentile, given the high diagnostic sensitivity and specificity of this pattern ^(5)^. Biochemical confirmation should include the 24h-UFC (adjusted for body surface area), low-dose (typically 1 mg) dexamethasone suppression test (LDDST), and late-night serum or salivary cortisol ^(10,11)^.

In children and adolescents, Cushing’s syndrome can be reliably excluded by two normal 24h-UFC results along with a normal LDDST. A midnight sleeping serum cortisol <1.8 µg/dL (<50 nmol/L) also excludes CD with 94-100% sensitivity and 100% specificity in pediatric patients, though hospital sampling is required ^(5)^. Late-night salivary cortisol (LNSC) may substitute for the midnight serum test ^(12,13)^, with sensitivity 93-100%, and specificity 95-100% ^(5)^; however, its use is limited by the absence of well-defined ageand assay-specific reference intervals ^(5)^. MRI identifies only 50-63% of pediatric corticotroph adenomas due to their very small size. If MRI is negative, inferior petrosal sinus sampling (IPSS) is strongly recommended, confirming a central ACTH source with nearly 100% sensitivity in experienced centers. A central-to-peripheral ACTH gradient ≥3 following corticotropin-releasing hormone (CRH) or desmopressin stimulation confirms CD, and lateralization accuracy varies from 58-87%, depending on the series ^(5)^.

First-line treatment is selective transsphenoidal adenomectomy performed by an experienced pediatric neurosurgeon ^(14)^. Second-line radiotherapy provides disease control in most children within 12-18 months but carries long-term risks of hypopituitarism ^(5,15,16)^. Steroidogenesis inhibitors (metyrapone, ketoconazole) are also regarded as second-line therapy, usually employed short-term to stabilize severe hypercortisolism or bridge to surgery. In children, long-term metyrapone suppository treatment has been associated with clinical and biochemical improvement without reported developmental adverse effects ^(17)^. Osilodrostat’s safety and pharmacological profile are currently being assessed in a small phase II study (NCT03708900), representing the first evaluation of this agent in pediatric patients (<18 years), with completion expected in 2025 ^(18)^. With specialized care, prognosis for pediatric CD is favorable. Early biochemical remission is associated with identifiable microadenomas and postoperative cortisol <1 µg/dL (28 nmol/L).

Long-term remission is highest in younger children and in those without cavernous sinus invasion ^(19)^. Nevertheless, even after remission, children with CD may experience persistent reductions in quality of life and neurocognitive functioning. Longitudinal research demonstrates residual impairments in attention, memory, and psychosocial adjustment after remission, suggesting that hypercortisolism during critical developmental periods may have lasting effects ^(20-23)^. Lifelong follow-up is necessary to address these neurocognitive sequelae as well as the risk of recurrence (6-40%), pubertal disturbances, and growth impairment ^(5)^. In children and adolescents, growth hormone (GH) testing should be performed 3-6 months post-remission, as early detection and treatment of GH deficiency are critical for optimizing catch-up growth and achieving target adult height. GH replacement in confirmed cases generally improves growth outcomes, albeit obesity may persist ^(24)^.

## PUBERTY/ADOLESCENCE (11-18 YEARS)

### Case vignette

A 15-year-old girl was referred for evaluation of obesity and menstrual irregularity. Over the previous two years, she had gained 15 kg and developed severe acne, purple striae, and depressive symptoms. Her height had plateaued, and she reported being bullied at school. Laboratory tests showed impaired glucose tolerance, elevated LNSC, increased 24h-UFC, and failure to suppress cortisol on LDDST. ACTH was elevated, with pituitary MRI showing a suspected left-sided 6-mm microadenoma. She underwent transsphenoidal surgery and developed transient postoperative corticotrope insufficiency. Over the subsequent year, she lost 7 kg, and her menses returned.

### Mini-review

The typical age of onset for pediatric-onset CD is 12-14 years, corresponding with puberty ^(8)^. The marked male predominance observed before puberty becomes less pronounced in midto late adolescence ^(5)^. Diagnosing Cushing’s syndrome in adolescents is particularly challenging, as many teenagers have overlapping features such as weight gain, acne, and mood changes. Distinguishing true CD from non-neoplastic hypercortisolism associated with obesity, depression, or chronic stress is therefore essential ^(6)^. In adolescents, the hallmark is weight gain with reduced growth velocity, often accompanied by changes in facial appearance, truncal obesity, violaceous striae, acne, virilization or menstrual disturbance, and psychological or academic difficulties ^(8)^. Pubertal timing may be abnormal in either direction: delayed puberty due to cortisol-induced hypogonadotropic hypogonadism, or, less commonly, androgen-driven premature adrenarche or peripheral precocious puberty resulting from ACTH-stimulated adrenal androgen excess ^(6,25)^.

Biochemical confirmation follows pediatric protocols: repeated 24h-UFC (adjusted for body surface area), assessment of diurnal cortisol rhythm (midnight serum or LNSC), and LDDST, interpreted against ageand assay-specific cut-offs after excluding exogenous glucocorticoid exposure ^(5)^. High-resolution pituitary MRI is recommended, although corticotroph microadenomas are often very small and may not be visualized in up to ~40-50% of pediatric CD ^(5)^. In adolescents with biochemically confirmed ACTH-dependent hypercortisolism and negative imaging, IPSS at specialty centers is recommended to confirm a pituitary source and, cautiously, assist with lateralization ^(5)^. Transsphenoidal surgery by an experienced pediatric neurosurgeon remains first-line therapy, offering high remission rates and low postoperative hypopituitarism ^(5)^.

Radiotherapy and medical therapy are reserved for persistent or recurrent disease, while bilateral adrenalectomy is considered solely for severe, refractory hypercortisolism or life-threatening situations ^(5)^. As with pediatric CD, lifelong follow-up is essential, as achieving biochemical remission does not equate to complete recovery. Management must extend beyond biochemical control to address catch-up growth, normalization of puberty and fertility, bone health, and the substantial psychosocial burden related to body image, mood, and academic performance ^(5,22,23,26)^. Ideally, these complex needs should be addressed within a structured multidisciplinary transition pathway to adult endocrine care ^(5,8)^.

## ADULT WOMEN

### Case vignette

A 32-year-old woman presented with progressive weight gain, oligomenorrhea, hirsutism, easy bruising, and uncontrolled hypertension. She reported two years of infertility and had been diagnosed with “polycystic ovary syndrome”. An X-ray performed for chronic back pain revealed an atraumatic L1 vertebral fracture. Biochemical evaluation showed a 24h-UFC six times the upper limit of normal, elevated LNSC, and non-suppressed morning serum cortisol after LDDST. ACTH was elevated, and pituitary MRI revealed a 6-mm right-sided microadenoma. IPSS confirmed a central-to-peripheral ACTH gradient ≥ 3 after desmopressin stimulation with right-sided lateralization. She underwent successful selective transsphenoidal adenomectomy but developed postoperative glucocorticoid withdrawal syndrome characterized by severe fatigue and myalgias. Glucocorticoid replacement was temporarily increased to supraphysiological doses and was then gradually tapered. She also received thromboprophylaxis with low-molecular-weight heparin, physiotherapy, and bisphosphonates. Over the following year, she experienced substantial clinical improvement, including restoration of regular menstrual cycles.

### Mini-review

In young and middle-aged women, CD is most commonly caused by ACTH-secreting pituitary microadenomas ^(1,2)^. Clinically, patients typically present with classic features of hypercortisolism including central obesity, facial plethora, easy bruising, menstrual irregularities, hirsutism, mood changes, osteoporosis, and cardiometabolic comorbidities ^(1)^. However, many of these signs are nonspecific and overlap with lifestyle-related conditions such as metabolic syndrome ^(27)^. Several scoring systems have been proposed to enhance pretest probability ^(28,29)^, but despite high specificity, their low sensitivity in external validation limits clinical utility ^(30)^.

Current guidelines recommend screening adults with age-inappropriate comorbidities (e.g., early osteoporosis or hypertension), those with multiple or progressive features of Cushing’s syndrome, and individuals with adrenal incidentalomas consistent with adenoma ^(11,31)^. Diagnostic evaluation relies on three first-line tests: LDDST, LNSC, and 24h-UFC, with at least two abnormal results needed to confirm the diagnosis ^(10,11)^. Notably, repeated testing (2-3 times) with 24h-UFC and LNSC may be required to detect or exclude Cushing’s syndrome, as cortisol concentrations demonstrate substantial interand intraindividual variation ^(32,33)^; the most extreme anomalies are seen in rarer, cyclic Cushing’s syndrome cases ^(34,35)^. High-resolution 3.0 and 7.0 Tesla MRI improves lesion detection, yet imaging remains negative or inconclusive in up to 27% of cases ^(36)^. In complex scenarios, molecular PET imaging may help localize the causative lesion ^(37,38)^.

In ACTH-dependent hypercortisolism, a pituitary macroadenoma confirms CD. If no lesion or a microadenoma-like lesion <6 mm is seen (findings occurring in up to 20% of the general population), further evaluation is required ^(39)^. IPSS remains the gold standard for distinguishing pituitary from ectopic ACTH secretion ^(10)^. The primary goals of CD treatment are remission of hypercortisolism, management of comorbidities and cardiovascular risk, restoration of hypothalamic-pituitary-adrenal axis function, preservation of fertility and pituitary function, and improvement of visual deficits in cases of suprasellar macroadenomas ^(40)^. Transsphenoidal surgery is the first-line treatment, while persistent or recurrent disease may require repeat surgery, radiotherapy, or medical therapies ^(10)^. These comprise steroidogenesis inhibitors (e.g., ketoconazole, levoketoconazole, metyrapone, and osilodrostat), pituitary-directed agents (e.g., pasireotide or off-label, cabergoline, with variable efficacy) ^(17)^, and, in selected scenarios, glucocorticoid receptor antagonists such as mifepristone ^(41-43)^. Bilateral adrenalectomy remains a definitive option for refractory disease or when rapid control of hypercortisolism is required ^(10)^. Postoperative management requires structured surveillance, as many patients experience prolonged morbidity even after biochemical remission. In the immediate postoperative period, morning cortisol and ACTH measurements are essential to confirm remission and guide glucocorticoid replacement ^(10)^.

Thromboprophylaxis using low-molecular-weight heparin is recommended (unless contraindicated) from diagnosis and continued for up to three months after biochemical cure, due to the sustained hypercoagulable state ^(44)^. During the first six months, clinical reassessment should address weight, blood pressure, mood, muscle strength, and fatigue, alongside evaluation of metabolic status and pituitary hormone axes ^(10,45)^. Glucocorticoid replacement must be tapered in line with hypothalamic-pituitary-adrenal axis recovery, with initially supraphysiological doses in the first weeks after surgery to manage glucocorticoid withdrawal symptoms ^(46)^. Lifelong follow-up is required, including annual biochemical screening for recurrence (LNSC or LDDST), and targeted management of cardiometabolic risk, bone fragility, cognitive dysfunction, and quality of life ^(10,45)^. Bone mineral density testing every 1-2 years is advised. Recurrence risk is highest within the first five years but persists lifelong, necessitating ongoing multidisciplinary follow-up ^(39,45)^.

Hypercortisolism substantially impacts social and occupational functioning: Large Scandinavian registry studies demonstrated reduced employment, increased sick leave and disability pensions up to six years before diagnosis and long after remission, with 20%-25% of patients requiring disability support ^(47,48)^. Long-term care should therefore address not only somatic health but also support functional recovery and reintegration into daily and occupational activities.

### Pregnancy in CD

Special considerations are required in certain circumstances such as pregnancy. Cushing’s syndrome during pregnancy is rare but carries significant maternal (hypertension, diabetes, preeclampsia, venous thromboembolism) and fetal risks (miscarriage, preterm birth, growth restriction) ^(49-51)^. Diagnosis is challenging due to physiological cortisol increases in pregnancy. The most reliable tests are 24h-UFC (≥ 3× ULN) and midnight serum cortisol, with MRI (without gadolinium) for imaging ^(50)^. Second-trimester surgery is the preferred treatment for both adrenal Cushing’s syndrome and CD. When surgery is not an option, metyrapone is the medical therapy of choice, although cabergoline may also be considered for CD ^(17)^. Pasireotide, levoketoconazole, and osilodrostat are contraindicated in pregnancy, with no safety data available. Mitotane is contraindicated due to teratogenicity and prolonged tissue persistence, requiring delayed conception after treatment. Mifepristone is contraindicated because of its antiprogestogenic effects, and there are no pregnancy data for relacorilant ^(17)^. Patients require intensive monitoring during pregnancy and structured cardiometabolic follow-up postpartum ^(49,50)^.

## ADULT MEN

### Case vignette

A 45-year-old man presented with resistant hypertension, new-onset type 2 diabetes, muscle weakness, and low libido. He reported easy fatigability and irritability. Physical examination revealed truncal obesity and mild facial plethora. Morning testosterone was low, with suppressed LH/FSH. Hypercortisolism was confirmed by elevated 24h-UFC, loss of salivary cortisol diurnal rhythm, and failure of morning serum cortisol suppression on LDDST. ACTH was elevated. MRI showed an 8-mm pituitary adenoma, and a desmopressin test confirmed a pituitary origin. He underwent transsphenoidal surgery. Postoperative adrenal insufficiency was managed with glucocorticoid replacement, with gradual recovery of testosterone levels.

### Mini-review

Cushing’s disease is less common in men than women (female-to-male ratio 4:1) ^(52)^, but when it does occur, it may manifest with more pronounced clinical features including striae, hypertension, myopathy, osteoporosis, and kidney stones ^(53,54)^. Moreover, secondary hypogonadism (manifested as low libido, erectile dysfunction, and reduced testosterone concentrations) affects 75%-93% of men at diagnosis of Cushing’s syndrome ^(55-58)^, and can serve as a surrogate marker of glucocorticoid excess ^(55)^. Diagnostic evaluation of CD follows the same algorithm as in women and therapeutic recommendations are also similar, with transsphenoidal surgery as first-line treatment ^(10,11)^. Most men recover from secondary hypogonadism during the first year after successful surgery ^(55-58)^. Thus, testosterone replacement may not be necessary at the time of Cushing’s syndrome diagnosis if surgery is planned; however, continued postoperative surveillance is essential for early detection of persistent or new hypogonadism after treatment (e.g., postsurgical panhypopituitarism), with testosterone therapy guided by symptoms, biochemical assessment, and current guidelines ^(59)^. In adults with CD, GH testing should typically be delayed for 1-2 years after remission, as GH secretion often recovers spontaneously over time ^(24)^. GH replacement may improve body composition, lipid profile, bone density, and quality of life, but requires individualized decision-making given potential adverse effects on glucose metabolism ^(24)^. The choice of steroidogenesis inhibitor should consider local availability and potential side effects, as hypogonadism may worsen with ketoconazole use ^(41)^. Further management should focus on impaired erythropoiesis ^(55,60)^, persistent cardiovascular risk, myopathy, osteoporosis, mental health, and structured rehabilitation for return to work and social reintegration.

## ELDERLY PATIENTS (≥ 65 YEARS)

### Case vignette

A 73-year-old woman was referred from geriatrics for refractory hypertension, recurrent fractures, sarcopenia with frequent falls, and poorly controlled diabetes. She was described as “frail,” exhibiting cognitive slowing and depression. Clinical examination revealed central adiposity, thin skin, multiple ecchymoses, and proximal myopathy. Bone densitometry showed osteoporosis with a T-score of -3.2. Hypercortisolism was confirmed by elevated LNSC and 24h-UFC. ACTH was elevated. MRI showed a 12-mm pituitary macroadenoma. Despite high surgical risk and progressive clinical decline, she underwent carefully planned transsphenoidal surgery. Postoperatively, mild hypercortisolism persisted due to residual tumor. Medical therapy with steroidogenesis inhibitors was initiated but discontinued because of side effects. The patient received bisphosphonates, physiotherapy, and psychiatric counseling.

### Mini-review

In older adults, the true prevalence of CD is likely underestimated, as many symptoms overlap with normal aging and common comorbidities. CD becomes less common as adrenal-dependent Cushing’s syndrome increases with age ^(7,61,62)^. In ERCUSYN, only ~10% of all Cushing’s syndrome patients and 8-9% of CD patients were ≥65 years ^(63)^. Aging also reduces the typical female predominance, with around 60% of elderly patients with CD being female. Older patients more often present with macroadenomas, including extrasellar lesions (~17%), while microadenomas are less frequent (~39%) ^(63)^. The clinical picture is less typical and is more driven by comorbidities. Compared with younger patients, elderly patients with Cushing’s syndrome report less weight gain and show fewer skin changes and hyperandrogenism-related signs such as hirsutism, hair loss, and reduced libido ^(7,63)^. In contrast, hypertension, diabetes, myopathy, fractures, cardiovascular disease, and thrombosis are more frequent ^(63)^.

Cognitive decline is also common, and may only partially improve after treatment, with age and prolonged cortisol exposure contributing to persistent memory deficits ^(64,65)^. Depression appears to be less frequently reported in the elderly ^(63)^, likely due to underrecognition, as late-life depression often presents with predominantly somatic rather than mood symptoms. 24h-UFC supports the diagnosis of CD slightly less often in older than younger patients (~87% vs ~95%), and renal impairment, polypharmacy, and chronic illness may further complicate interpretation ^(7,63)^. Consequently, a high index of suspicion is required in older patients with new or worsening hypertension, diabetes, fractures, or unexplained muscle weakness, even in the absence of overt Cushingoid features. Invasive diagnostic and therapeutic procedures should be weighed cautiously in very frail patients, balancing potential benefit and risk. Management requires an individualized approach that considers surgical risk, life expectancy, and symptom burden. Transsphenoidal surgery remains first-line therapy but is used more selectively with age.

In ERCUSYN, ~31% of elderly CD patients never underwent surgery (vs ~11% of younger patients), with first-line medical therapy and/or radiotherapy chosen more frequently ^(63)^. In expert centers, elderly patients had acceptable complication rates but experienced lower remission after TSS (≈52% vs 65%) and shorter disease-free survival ^(63)^. To our knowledge, no large studies specifically address medical therapy in elderly CD patients; available data are from adult cohorts encompassing a broad age range, with outcomes rarely reported separately for older individuals. In clinical practice, agents such as metyrapone are generally considered effective in older patients ^(17)^, but safety and tolerability must be assessed individually given comorbidities and polypharmacy. Overall mortality is higher in the elderly, and older age is an independent predictor of death, mainly due to vascular events, infections, and tumor progression ^(63)^. Ongoing management should prioritize functional status, prevention of falls and fractures, cardiovascular risk reduction, and optimization of quality of life.

## CONCLUSIONS

Cushing’s disease presents and progresses differently across the lifespan, leading to age-specific diagnostic challenges and requiring individualized therapeutic approaches. Symptoms frequently overlap with common developmental or age-related conditions, contributing to delays in diagnosis. Careful attention to age-related clinical features and test interpretation is crucial for accurate evaluation and tailored management. Mortality risk in CD varies with age but remains closely linked to persistent hypercortisolism and its systemic complications.

Mortality is generally low in pediatric Cushing’s syndrome (≈2.5% over 2-4 years)most often related to sepsis ^(66)^ but children and adolescents remain susceptible to long-term cardiometabolic consequences that may persist into adulthood. In adults, excess mortality is well documented and is primarily attributable to cardiovascular disease, infections, and persistent hypercortisolism. In a nationwide cohort study with a median follow-up of 10.6 years, 18% of patients with CD died compared to 9% of matched controls (HR 2.1), with markedly worse outcomes in those who did not achieve remission ^(67)^. Mortality risk persists even among patients in remission, as observed in large contemporary cohorts ^(68)^, underscoring the importance of long-term management of comorbidities. Across all age groups, such management should be coordinated within a multidisciplinary framework that reflects the patient’s developmental stage, comorbidities, and long-term risk profile. Recognizing these age-dependent distinctions is essential to support earlier detection and improve outcomes across the lifespan.
